# Analysis of Hair Tonics and Restoratives

**Published:** 1871-06

**Authors:** 


Analysis of Hair Tonics and Restoratives.—The following are the results obtained by Prof. C. F. Chandler, from the analysis of fifteen so-called hair tonics and restoratives.

| | Grains of Lead in one fluid ounce. |
|---|---|
| Clark's distilled Restorative for the Hair, | 0 11 |
| Chevalier's Life for the Hair, | 1 02 |
| Circassian Hair Rejuvenator | 2 71 |
| Ayer's Hair Vigor, | 2 89 |
| Wood's Hair Restorative, | 3 08 |
| O'Brien's Hair Restorative of America, | 3 28 |
| Gray's celebrbted Hair Restorative, | 3 39 |
| Phalon's Vitalia, | 4 69 |
| Ring's Vegetable Ambrosia, | 5 00 |
| Mrs. S. A. Allen's World Hair Restorer, | 5 57 |
| L. Knittels' Indian Hair Tonique, | 6 29 |
| Hall's Vegetable Sicilian Hair Renewer, | 7 13 |
| Tebbbetts' Physiological Hair Regenerator, | 7 44 |
| Martha Washington Hair Restorative, | 9 80 |
| Singer's Hair Restorative, | 16 39 |

